# Virtual training and technical assistance: a shift in behavioral health workforce access and perceptions of services during emergency restrictions

**DOI:** 10.1186/s12909-022-03598-y

**Published:** 2022-07-27

**Authors:** Kristen G. Powell, Michael J. Chaple, Maxine Henry, Cory Morton, Sara J. Becker, Heather J. Gotham, Holly N. Hagle, Ashley C. Helle, Laurie J. Krom, Rosemarie Martin, Todd D. Molfenter, Nancy Roget, Beth A. Rutkowski, Isa I. Velez-Echevarria, Ruth Yanez, Cross-Technology Transfer Center (TTC) Workgroup on Virtual Learning, Kristen G. Powell, Michael J. Chaple, Maxine Henry, Cory Morton, Sara J. Becker, Heather J. Gotham, Holly N. Hagle, Ashley C. Helle, Laurie J. Krom, Rosemarie Martin, Todd D. Molfenter, Nancy Roget, Beth A. Rutkowski, Isa I. Velez-Echevarria, Ruth Yanez

**Affiliations:** 1 Rutgers University School of Social Work, New Brunswick, NJ USA; 2 New York State Psychiatric Institute, New York, NY USA; 3 National Latino Behavioral Health Association, Peña Blanca, NM USA; 4 Brown University School of Public Health, Providence, RI USA; 5 Stanford University School of Medicine, Palo Alto, California USA; 6 University of Missouri-Kansas City School of Nursing and Health Studies, Kansas City, MO USA; 7 Department of Psychological Sciences, University of Missouri, Columbia, MO USA; 8 University of Wisconsin-Madison, Madison, WI USA; 9 University of Nevada, Reno, Reno, Nevada USA; 10 UCLA Integrated Substance Abuse Programs, Los Angeles, California USA; 11 Universidad Central del Caribe, Bayamon, PR USA

**Keywords:** COVID-19, Behavioral health, Training, Workforce development

## Abstract

**Background:**

To respond to the COVID-19 pandemic, the Substance Abuse and Mental Health Services Administration-funded Technology Transfer Centers had to rapidly adapt to ensure that the behavioral health workforce had continuous access to remote training and technical assistance (TTA). Although the Technology Transfer Centers have historically relied partially upon virtual methods for delivering TTA, the shift to a strictly virtual approach necessitated by COVID-19 restrictions has raised new questions for how to best proceed with services when social distancing guidelines are relaxed. The objective of this exploratory paper was to compare TTA provision in the six-month period prior to (9/1/19 thru 2/28/20) and during (4/1/20 thru 9/30/20) early COVID-19 restrictions to determine the extent to which the shift to virtual service provision impacted the behavioral health and medical workforce. Specifically, we examined participants’ access to TTA, geographic reach of TTA, and workforce perceptions of satisfaction and utility with TTA provision.

**Method:**

Participant and event-level data were analyzed to compare the following metrics before and during the COVID pandemic: number of events and attendees; participant demographics; zip codes reached; coverage of rural, suburban, and urban areas; and perceptions of satisfaction with and utility of training.

**Results:**

Findings showed a 40% increase in the number of events delivered (*p < .001*) and a 270% increase in the number of attendees *(p < .001*) during the COVID period when TTCs relied exclusively on virtual delivery. Geospatial analyses linking zip codes to a schematic of rural, suburban, and urban classifications throughout the United States revealed significant increases in the number of zip codes reached during the COVID time period. Satisfaction levels were comparable before and during the pandemic.

**Conclusions:**

Findings show that expanded access to TTA services via virtual formats resulted in reach to more diverse attendees and regions, and did not come at the expense of satisfaction. Results suggest that virtual TTA should continue to be an important component of TTA offerings post-pandemic.

## Background

The coronavirus 2019 (COVID-19) pandemic has radically changed almost all facets of our lives and the behavioral health workforce was not immune to these changes. Behavioral healthcare providers had to rapidly shift, essentially overnight, to virtual service delivery in a way that ensured minimal service disruption for those in care [1, 2]. Providers were also challenged to engage a growing number of new patients remotely, as behavioral health needs surged in the early days of the pandemic [3]. Accumulating evidence has documented pandemic-related increases in both recurrence and initiation of mental health symptoms and substance use, both of which have increased the number of individuals requiring behavioral health services since the onset of the pandemic [4, 5]. In addition to professional stressors brought on by the pandemic, providers have also had to cope with personal stressors including the threat to themselves or family members of contracting the virus [1]. In shifting from a predominantly in-person service delivery model to a virtual model, the workforce has therefore required additional support to effectively adapt protocols for virtual delivery and to promote best-practices for staff wellness and self-care [6, 7]. Across the globe, education and workforce training programs made immediate shifts to virtual and distance learning environments to support behavioral health and medical students and professionals [8–11].

Over the past 25 years the Substance Abuse and Mental Health Services Administration (SAMSHA) has provided cutting edge training and technical assistance (TTA) through their Addiction Technology Transfer Centers (TTCs) [12]. A network of Addiction TTCs was established in 1993 to provide locally responsive training and technical assistance to the front-line addiction treatment and recovery workforce. In 2018, SAMSHA added Mental Health TTCs and Prevention TTCs, focused on providing comprehensive education, training and supports for providers in the areas of mental health and prevention, respectively [13]. Each of the three Technology Transfer Center (TTC) networks is comprised of ten regional centers, two national focus centers for special populations (i.e., National Hispanic and Latino, National American Indian and Alaska Native) and a network coordinating office. Together the TTC Networks provide TTA services to all US states, Freely Associated States, and territories. The specific charge of the TTC Networks is to ensure the modernization of the behavioral health service system, by building the capacity of the behavioral health workforce to provide evidence-based interventions via locally and culturally responsive TTA [14].

Prior to the pandemic, the TTCs offered in-person and virtual TTA in three categories: basic, targeted, and intensive [15]. Basic TTA focuses on information dissemination to a broad, heterogenous audience and consists of brief consultation, mass mailings, publications, e-newsletters, websites, social media, and single-event webinars. Targeted TTA enhances practitioners’ readiness and builds the capacity to implement evidence-based practices in a specific setting or context. Targeted TTA is commonly offered via online courses, webinar series, communities of practice, and other short-term training series. Intensive TTA supports full incorporation of an innovation or practice into real-world settings that requires changes in policies, practices, and system functioning. The TTCs offer assistance based on principles of both “push” and “pull” demand [16]. The TTCs may “push” TTA by offering events for their local community based on annual needs assessments and feedback from regional advisory boards, or TTA can be “pulled” via request by specific behavioral health organizations. When organizations request TTA, the provision of training and education is ultimately driven by an implementation plan that reflects mutually agreed-upon goals, roles, and responsibilities between the TA provider and recipient [17].

Similar to behavioral healthcare providers, the TTCs suspended in-person service provision during the pandemic and rapidly transitioned TTA across the continuum (e.g., basic, targeted, and intensive) to virtual, with every attempt made to limit the disruption of services and dilution of technology transfer activities [15]. In doing so, TTCs met providers where they were, to address the rapidly emerging need for information, guidelines, tools and evidence-based practices that could prepare the behavioral healthcare workforce to effectively assist the communities they serve. TTA topics were immediately adapted to include information on COVID-19, its impact on services and those receiving care, and to provide guidance on how to effectively navigate this new service environment.

## Present study

Although the TTCs have historically relied in part upon virtual methods for delivering TTA, the shift to a strictly virtual approach necessitated by the COVID-19 pandemic has raised new questions for how to best proceed with TTA provision as social distancing guidelines are relaxing. A recent survey of TTC Directors from all three networks (Addiction, Mental Health, and Prevention) identified a common perception that TTA delivered virtually offered significant advantages, particularly with regard to its ability to expand provider access to TTA services and activities [10]. These perceptions merit more rigorous investigation to explore the extent to which TTA delivered virtually actually impacts access to and the quality of these services, in order to inform potential hybrid models for TTA once pandemic restrictions are lifted.

The objective of this exploratory study was to compare the reach of and engagement in TTC events prior to and during the early months of the pandemic, as well as participant perceptions of satisfaction with and utility of TTA. Specifically, this study compared participant and event-level data that reflected TTA provision in the six-month period prior to (September 2019 thru February 2020) and the six-month period during (April thru September 2020) the declaration of COVID-19 as a US national emergency and the implementation of social distancing requirements [18]. Reach was explored primarily in terms of the number of participants engaged in TTA activities as well as the geographic location of engaged participants using geographic information systems methods. Satisfaction was assessed with regard to the quality of instruction, the benefit of content to participants’ work, usefulness of materials, and willingness to recommend the event to a colleague. In addition, we examined whether there were any differences with regard to access to and satisfaction with TTA services based on factors such as participant demographics, professional discipline, and employment setting. By comparing reach and satisfaction with TTA events over these two time frames, we aim to produce data that can inform efforts to educate the behavioral health workforce post-pandemic.

## Methods

### Study context and procedures

In accordance with SAMHSA funding requirements, participant-level data are collected using the Government Performance and Results Act (GPRA) tool for participants who attend TTA services provided by TTCs [19]. These instruments are approved by the US Office of Management and Budget and help to determine the reach, consistency, and quality of the TTC program and do not collect personally identifiable information. The same GPRA instruments are used across the three TTC networks for any type of TTA event.

This study used data collected from two GPRA forms: 1) *Event Description (ED) Form*, an event-level form; and 2) *Post Event Form*, a participant-level form. The *Event Description Form* is completed by the sponsoring TTC and includes details on the event, including total number of participants in attendance. The *Post Event Form* is collected from participants within 7 days of the TTA service and assesses information on the participants’ demographics and satisfaction with the event. Attendance at TTA events and completion of the *Post Event Form* is completely voluntary. Not all attendees complete the *Post Event Form*, therefore the number differs from total numbers of participants in attendance.

Permission was sought from all 39 TTCs to include their GPRA data in this analysis, of which 38 (97%) agreed to be included. All participant level data are anonymous, and the secondary use of these de-identified data is considered either ‘exempt’ or ‘not human subjects’ by individual authors’ corresponding Institutional Review Boards. GPRA data for the TTCs were downloaded and aggregated for two time periods: 1) the six-month period prior to when COVID-19 restrictions were implemented, September 1, 2019 thru February 28, 2020 (“pre-COVID”); and 2) the six-month period following implementation of COVID-19 restrictions, April 1, 2020 thru September 30, 2020 (“during-COVID”). For both periods combined, data collected through the *Event Description Form* contained information for a total of 2257 events and 175,766 participant attendees. Data collected through the *Post Event Form* included a total of 85,528 (49% response rate) unique participant responses across the 50 US states, the District of Columbia, five US territories (American Samoa, Commonwealth of the Northern Mariana Islands, Guam, Puerto Rico, and the US Virgin Islands), and three Freely Associated States (the Republic of the Marshall Islands, the Republic of Palau, and the Federated States of Micronesia). See Table 1 for breakdown of event and participant data collected for the pre-COVID and during-COVID timeframes.

**Table 1:** Descriptive Technology Transfer Center (TTC) event data

Variable	Pre-COVID	During-COVID	t-value (probability)	Effect Size
Number of events^a^	939	1318	–	
Number of participants attended^a^	37,363	138,403	–	
Number of GPRA surveys^b^	20,568	64,960		
	Mean (SD)	Mean (SD)		
Number of participants per zip code^b^	1.77 (4.50)	5.67 (10.47)	t = − 43.91 (*p* < .001)	*d* = .48
Participants per event^c^	39.79 (55.95)	105.01 (146.90)	t = −14.69 (*p* < .001)	*d* = .69
Contact Hours per event^a^	4.24 (4.82)	2.76 (4.06)	t = 7.70 (*p* <.001)	*d* = .36

^a^Gathered from Event Description Forms (ED Forms)

^b^Number of Post-Event GPRA evaluation forms collected during each period

^c^Reported on ED Forms based on number of participants in attendance (not number of GPRA collected)

### Data variables

Data extracted from the *Event Description Form* included the date on which the event was delivered, event length, the number of participants in attendance, and the number of continuing education hours granted to participants for each event. Data extracted from the *Post Event Form* included participant level variables such as demographics (e.g., race, gender, education level), professional discipline, employment setting, and zip code of employment setting. In addition, the *Post Event Form* included four items to measure participant satisfaction with TTA events. These questions included one item about overall satisfaction (i.e., “How satisfied were you with the overall quality of this event?”) rated on a 5-point Likert type scale (*very dissatisfied* to *very satisfied)*, and two items asking participants to indicate their level of agreement on a 5-point Likert type scale (*strongly agree* to *strongly disagree*) related to how the TTC event will help in their profession (i.e., “I expect this event to benefit my professional development and/or practice,” and “I will use the information gained from this event to change my current practice”). The fourth item indicated (*yes*/*no*) whether participants would recommend the training to a colleague.

### Statistical analysis

GPRA data were downloaded into Microsoft CSV files and then exported into SPSS version 25 for data analyses. Analyses included descriptive statistics to compare trends across the two time periods (i.e., pre- and during-COVID-19 restrictions). This included cross-tabulations for nominal and ordinal level data and comparison of means for interval level data. Tests were conducted to determine the statistical significance of observed changes, chi-square for cross-tabulations, and t-tests for comparisons of means between pre- and during-COVID averages.

Because of the extremely large sample size, we calculated effect sizes to ensure that we did not only consider statistical significance (which is highly likely in a large sample), but also considered the size of the effects. Cohen’s *d* was calculated to determine the effect sizes of mean comparisons using the pooled variance estimates, with values interpreted according to the guidance of Cohen such that 0.20 indicated a small effect, 0.50 indicated a moderate effect, and 0.80 indicated a large effect [20]. Phi (used for 2 × 2 contingency tables) and Cramer’s V coefficients were also computed as an adjustment of chi-square significance to account for large sample sizes [21]. Cramer’s V values were interpreted using the following ranges: 0–0.05 = no or very weak association; > 0.05 = weak association; > 0.10 = moderate association; > 0.15 = strong association [21].

Additionally, ArcMap 10.8.1 software was used to show the reach of trainings pre- and during COVID-19 by mapping zip codes in the US States, Freely Associated States, and territories where TTC training participants were located into three mutually exclusive groups: 1) zip codes where participants received TTA pre-COVID only; 2) zip codes where participants received TTA pre- and during-COVID; and 3) zip codes where participants received TTA during COVID only. In addition, using guidance from Hailu and Wasserman [22], participant zip codes were categorized as urban, suburban, or rural using the zip code level Rural-Urban Commuting Area Codes classification system [23]. Rural-urban commuting area codes classify US zip codes into metropolitan, micropolitan, small town, and rural areas using US census data on population density, urbanization, and commuting patterns [23]. Descriptive analyses and paired samples *t* tests were used to describe changes in the numbers of participants in urban, suburban, and rural zip codes.

## Results

Results in Table 1 demonstrate that in the six months during-COVID when services were being delivered exclusively via virtual formats, there were substantial increases in the number of TTA events delivered (+ 40%), the number of participants attending these events (+ 270%), and the number of GPRA evaluations collected (+ 216%) compared to the pre-COVID time period. Furthermore, there was a significant increase in the average number of participants in attendance at each event (40 vs. 105; + 163). Effect size calculations adjusting for sample size indicated that this increase was moderate in size. There was also a small to moderate decrease in the average duration of each TTA event from over 4 hours to under 3 hours (− 35%).

**Fig. 1:**
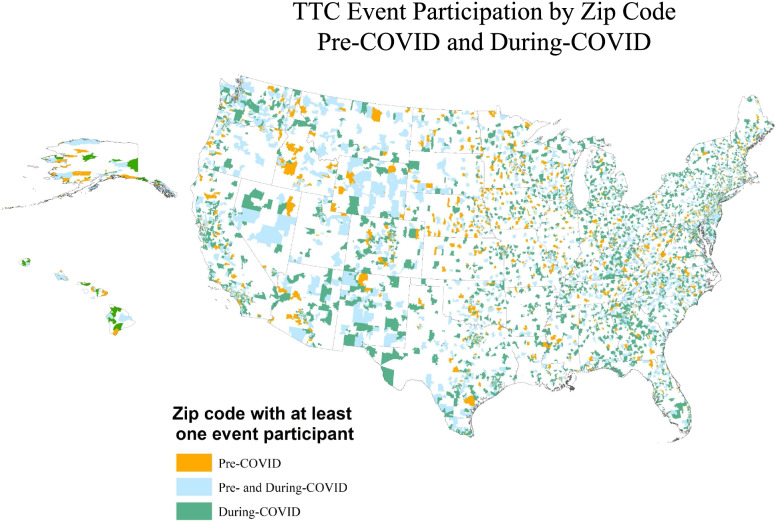
Zip Codes of Technology Transfer Center (TTC) Event Participants’ Workplace. Note: Analysis includes participants’ zip codes from all 50 U.S. states, the District of Columbia, and eight U.S. Territories and Freely Associated States (American Samoa, Commonwealth of the Northern Mariana Islands, Federated States of Micronesia, Guam, Puerto Rico, Republic of the Marshall Islands, Republic of Palau, and the U.S. Virgin Islands); however, due to the smaller geographic size of the Territories and Freely Associated States, they could not be adequately depicted on this map

Figure 1 provides additional data and context on the reach of TTA events with respect to participants’ employment location. Zip codes from all 50 US States, the District of Columbia, and eight Freely Associated States and territories were included in all analyses; however, due to the smaller geographic size of the territories, they could not be adequately depicted in Fig. 1. Areas of the map marked in orange illustrate zip codes of those who participated in TTA activities prior to COVID only *(n =* 1504 zip codes*)*, meaning there were zero individuals participating in TTA events in these zip codes during COVID restrictions. Areas of the map marked in green illustrate zip codes of those who participated in TTA activities during the COVID restrictions period only (*n* = 5556 zip codes), meaning there were zero individuals participating in TTA events in these zip codes during the pre-COVID period. Areas of the map marked in blue illustrate zip codes where individuals participated in TTA events during both time periods (*n* = 4171 zip codes). Closer examination of the map indicates that although there were many repeat participants for these time periods, a larger number of new participants in TTC services were engaged during the COVID restrictions period. Although not depicted in Fig. 1, the eight US Territories and Freely Associated States also experienced new or sustained zip code area participation during-COVID.

Additional paired samples t-test analyses linking zip codes to a schematic of rural, suburban, and urban classifications revealed that each type of region saw significant increases in the number of participants in the during-COVID time period. The mean number of participants per zip code increased from 2.02 to 6.89 (*t* = − 38.76, *p* < .001) in urban areas, from 1.10 to 2.84 (*t* = − 7.05, *p* < .001) in suburban areas, and from 1.33 to 3.79 (*t* = − 21.12, *p* < .001) in rural areas. Lastly, among the 5556 zip codes that saw new participants during-COVID, 64% were urban, 10.9% were suburban, and 25.1% were rural. Comparatively, zip codes that had any participation pre-COVID (*n* = 5675) had a geographic composition of 68.7% urban, 7.4% suburban, and 23.8% rural.

Table 2 offers a comparison of participant demographics for those attending TTA events in the pre- and during-COVID time periods. Chi-square results indicate a significant change in the composition of participant demographics during the two time periods with regard to gender, race/ethnicity, and education level. Here, results show an increase in participation by women (and a corresponding decrease in participation by men), an increase in participation by African Americans and Hispanics/Latinos (and a corresponding decrease in participation by Whites), and an increase in participation by master’s level individuals (and a corresponding decrease in participation by individuals with a high school degree, GED, or some college). Cramer’s V coefficients computed for each chi-square analysis in Table 2 reveal weak associations, suggesting that these changes in participant demographics were very small.

**Table 2:** Technology Transfer Center (TTC) event participant demographics and profession

Variable	Pre-COVID		During COVID		Sig	
	N	%	N	%	X^2^	Coeff^a^
**Gender**					286.43	.058
Male	4271	20.8	10,192	15.7	*p* < .001	*p* < .001
Female	16,175	78.6	54,355	83.7		
Transgender/other	122	0.6	413	0.7		
**Race/Ethnicity**						
Black/African American	2572	12.8	10,420	16.2	545.59	.080
Asian	372	1.8	1698	2.6	*p* < .001	*p* < .001
White	12,883	64.0	35,355	55.1		
Hispanic/Latino	2671	13.3	11,161	17.4		
AI/AN^b^	622	3.1	2104	3.3		
NH/PI^b^	167	0.8	410	0.6		
Multiracial	849	4.2	2979	4.6		
**Highest Degree Received**						
Less than high school	33	0.2	36	0.1	216.20	.050
HS/GED/Some college^b^	2078	10.2	4980	7.7	*p* < .001	*p* < .001
Assoc/Bach degree	6438	31.7	20,649	32.0		
Master’s degree	9798	48.2	33,500	51.8		
Doctoral degree	1609	7.9	4292	6.6		
Other	362	1.8	1154	1.8		
**Professional Discipline**						
Counselor	3215	16.4	12,234	19.7	1353.88	.129
Addictions professional	1602	8.2	4287	6.9	*p* < .001	*p* < .001
Psychiatrist/Psychologist	932	4.7	3008	4.8		
Social worker	3113	15.9	14,713	23.7		
Recovery/peer specialist	882	4.5	3293	5.3		
Criminal justice professional	238	1.2	725	1.2		
CHW^b^ /health educator	3432	17.5	7897	12.7		
Public/Business administrator	708	3.6	1354	2.2		
Researcher	344	1.8	548	0.9		
Medical professional	966	4.9	2073	3.3		
Student	881	4.5	1490	2.4		
Other	3309	16.9	10,579	17.0		
**Principal Employment Setting**						
SUD treatment program	2222	11.5	6015	9.7	903.56	.105
SUD prevention program	1086	5.6	5325	8.6	*p* < .001	*p* < .001
Recovery support program	422	2.2	1347	2.2		
MH treatment program	2459	12.7	11,526	18.5		
Trans. living/group home	252	1.3	578	0.9		
Health center/PC^b^ practice	1780	9.2	5549	8.9		
Hospital/skilled nursing	1119	5.8	2434	3.9		
Criminal justice/corrections	590	3.0	1842	3.0		
Education	4775	24.7	11,977	19.2		
Community based org	1295	6.7	5147	8.3		
Community coalition	439	2.3	1334	2.1		
Other	2916	15.1	9168	14.7		

^a^Phi (used for 2 × 2 contingency) and Cramer’s V adjust X^2^ significance for sample size

^b^*AI/AN* American Indian/Alaska Native, *NH/PI* Native Hawaiian/Pacific Islander, *HS* High school, *GED* General educational development, *CHW* Community Health Worker, *PC* Primary Care

Table 2 also offers a comparison across professional discipline and principal employment setting for those attending TTA events in the pre- and during-COVID time periods with chi-square results indicating a significant change in the composition of both. With regard to professional discipline, results show an increase in participation most noticeably by social workers and to a lesser extent by counselors. Additionally, there was a corresponding decrease in participation most noticeably by community health workers and health educators and to a lesser extent by addictions professionals, medical professionals, students, and business administrators. With regard to principal employment setting, results show an increase in participation most noticeably by those working in a mental health treatment setting and to a lesser extent by those working in substance use prevention programs and community-based programs; there was also a corresponding decrease in participation most noticeably by those working in education settings and to a lesser extent those working in substance use treatment programs and hospitals/skilled nursing facilities. Cramer’s V coefficients were again computed as an adjustment of chi-square significance for large sample sizes: results indicated that these changes in participant education and workplace setting were moderate in size.

Table 3 examines overall participant satisfaction with the TTA events, perceived utility of TTA events for professional practice, intent to use information from the event, and willingness to recommend the TTA events to a colleague, comparing pre- and during-COVID periods. For all four items, results show statistically significant increases from the pre-COVID period to the during-COVID period. However, the Cramer’s V coefficients, which adjusts chi-square significance for sample size, indicate that these differences were very weak. Therefore, results revealed that high ratings of satisfaction, quality, and usefulness of the material remained essentially unchanged during the COVID-19 period for the adapted TTA virtual delivery format.

**Table 3:** Technology Transfer Center (TTC) event participant satisfaction

Variable	Pre-COVID		During COVID		Sig	
	N	%	N	%	X^2^	Coeff*
**Satisfied Overall Quality of Event**						
Very satisfied	12,028	59.7	39,847	62.4	98.19	.034
Satisfied	6919	34.3	21,099	33.1	*p* < .001	*p* < .001
Neutral	916	4.5	2202	3.4		
Dissatisfied	161	0.8	325	0.5		
Very dissatisfied	121	0.6	362	0.6		
**Benefit Professional Practice**						
Strongly agree	11,294	56.1	36,117	56.7	45.07	.023
Agree	7499	37.3	24,077	37.8	*p* < .001	*p* < .001
Neutral	1104	5.5	3045	4.8		
Disagree	142	0.7	276	0.4		
Strongly disagree	83	0.4	197	0.3		
**Will Use Information from Event**						
Strongly agree	9415	46.9	31,880	50.2	119.90	.038
Agree	7852	39.1	24,195	38.1	*p* < .001	*p* < .001
Neutral	2415	12.0	6633	10.4		
Disagree	298	1.5	605	1.0		
Strongly disagree	90	0.4	205	0.3		
**Willing to recommend event to colleague**	19,244	96.5	62,561	97.7	92.98 *p* < .001	.033 *p* < .001

*Phi (used for 2 × 2 contingency) and Cramer’s V adjust X^2^ significance for sample size

## Discussion

This exploratory study found that during COVID-19 restrictions, the TTCs shift to virtual delivery enabled them to deliver far more TTA events to many more participants. Women and individuals who identify as BIPOC (Black, Indigenous, People of Color), specifically those identifying as African American and Hispanic/Latinx, participated in a greater number of TTA events as a result of the transition to virtual services during the COVID-19 pandemic, though these changes had small effect sizes. Further, this study found significant, moderate increases in participants accessing TTC services from urban, suburban, and rural employment areas. The TTCs experienced increased numbers of participants from mental health, substance use, and community-based employment settings during the pandemic, indicating the continued need for behavioral health training despite social distancing restrictions.

Prior publications by the TTC networks have found that a) TTC directors viewed the shift to virtual service provision as generally advantageous [15], and b) TTA events in the early months of the pandemic covered a range of topics including: racial equity; behavioral health needs; provider self-care; shifting to telehealth; evidence-based practices; networking; changing laws and policies; and organizational management and communication [24]. Many of these topics were commonplace prior to the pandemic, though interest in several of the topics (e.g., racial equity, shifting to telehealth, changing laws and policies, networking) surged in the early months of the pandemic [24]. The current study extended this prior work and demonstrated that the reach of these TTA events increased dramatically, in terms of the number of attendees and regions engaged, and the number of sessions offered, during the early months of the pandemic.

Most importantly, TTC participants’ perceptions of usefulness of and satisfaction with events delivered during pandemic restrictions were just as highly rated as compared to events delivered during the pre-COVID timeframe. Built on a model of technology transfer that emphasizes understanding the context in which promising practices are implemented [8], the TTCs were in a unique position to rapidly assess behavioral health workforce needs stemming from the pandemic restrictions and implement TTA to meet these emergent needs. This study showed that the TTCs were able to use their technology transfer model to adapt virtually to emerging needs and increase geographic reach without compromising the high satisfaction with TTA services.

Results of this study are generally consistent with prior international work documenting the benefits of virtual training as part of behavioral health and medical education. A 2020 study by Sadek and Kora described how medical schools in Egypt had to rapidly adopt online education and training during the early months of the pandemic, and found that students reported high satisfaction with virtual training with no complaints about the quality of instruction [25]. Similarly, Khursid and colleagues’ rapid narrative review of 19 studies argued that rather than viewing the pandemic as a disruption to education and training, it should be considered an opportunity to improve distance learning techniques and enhance educational delivery after the relaxation of social distancing orders [11]. International medical training programs have also cited the rise in virtual education as one of the few positive outcomes of the pandemic [26].

## Limitations

This study has a few limitations that should be considered. First, self-reported satisfaction does not account for actual behavioral outcomes, such as the ability to implement new skills on the job. Future studies might examine how TTC event participants were able to integrate new skills within their work. Second, we note that the GPRA response rate might have led to sample bias. Third, we are able to document shifts in reach and satisfaction, but are not able to make causal inferences as to why these changes occurred. Lastly, we acknowledge that attending any of the TTC virtual events could be impacted by access to or affordability of broadband [27] in homes during COVID-19 restrictions. Therefore, this study might contain some sample bias since those able to attend TTA services and complete satisfaction surveys would be contingent on their broadband access. Importantly, future work should consider who within the behavioral health workforce are being excluded by exclusively virtual TTA formats.

## Conclusions

Notwithstanding these limitations, this study ultimately revealed that the TTC networks were able to flexibly adapt to the global pandemic through virtual programming without compromising the high level of satisfaction and perceived benefit of TTC programming. At a time in which COVID-19 impacted all dimensions of personal and professional life [1], the TTCs supported behavioral health workforce development through TTA events delivered virtually and tailored to emerging needs. The fact that the TTCs were able to increase the number of events and number of attendees was not surprising – yet when combined with data suggesting that the geographic coverage of events increased, provider demographics shifted, and satisfaction was unchanged – the picture becomes far more encouraging. Results from this study suggest that TTCs can continue to expand access to TTA services for behavioral health professionals in order to potentially increase geographic reach without compromising participant satisfaction with the service provided. These findings lend guidance to TTCs and other behavioral health workforce development planning efforts to inform hybrid models of TTA delivery that could more routinely incorporate virtual delivery methods once the US and other countries have moved beyond the pandemic emergency restrictions.

## Data Availability

The datasets analyzed during the current study are not publicly available due permission limited to authors’ use but could become available from the corresponding author on reasonable request.

## Abbreviations

COVID-19: Coronavirus 2019
GPRA: Government Performance and Results Act
SAMHSA: Substance Abuse and Mental Health Services Administration
TTA: Training and technical assistance
TTC: Technology Transfer Center
